# Supplementary material for: Evaluating retinal thickness classification in children: A comparison between pediatric and adult optical coherence tomography databases
Source: PLoS One. 2024 Dec 30;19(12):e0314395. doi: 10.1371/journal.pone.0314395 (PMC11684601; doi:10.1371/journal.pone.0314395)
Supplement: Attachment — Submitted filename: Response to Editor_updated.pdf [file pone.0314395.s001.pdf]

PONE-D-24-23425: Point-by-point response

Response to Editor

1. Title needs to be reframed.

   Our response: the title has been reframed as

   "Evaluating Retinal Thickness Classification in Children: A Comparison Between Pediatric and Adult Optical Coherence Tomography Databases"

2. Abstract has plenty of sentence formation errors which necessitates it to be rewritten.

   Our response: Abstract has been rewritten.

3. "There is a tendency to overestimate the thinness of the macular, GCC, and GCIPL in children when using databases currently included in the SD-OCT." This sentence in the Conclusion section needs to be reframed.

   Our response: the sentence has been reframed as

   "When using the inbuilt adult database in the SD-OCT as a reference, there is a tendency to miss abnormal RNFL measurements but overestimate the thinness of the macular, GCC, and GCIPL in children."

4. Please review your reference list to ensure that it is complete and correct.

   Our response: Reference list has been corrected accordingly.

Response to Reviewer #1

1. It would be useful if the macular thickness association with the spherical equivalent was presented in tabular form for ease of understanding and better correlation with degree of refractive error. the data needs better presentation.

   Our response: The table has been added as

   Line 238: Table 4. Correlation between macular parameters with age and SE

2. The topic of RNFL and macular thickness in pediatric population has been well researched, your study is well done but it would be even more useful if these measurements were analysed for specific population and a detailed correlation with spherical equivalent was provided

   Our response: Correlation with spherical equivalent by SE groups has been added for

   RNFL

Line 192-193: Correlations between SE groups and RNFL thickness are shown in Table 2, where a significant correlation was found only in the myopic SE group.

Line 194: Table 2. Correlation between RNFL thickness with SE groups

Macular

Line 243-245: Statistically significant positive correlations were found in myopic SE for all the macular parameters except minimum foveal and centre ETDRS thickness. No significant correlation was found between the emmetrope and hyperope groups, as shown in Table 5.

Line 246: Table 5. Correlation between macular parameters with SE groups

GCC & GCIPL

Line 290-295: A significant weak positive correlation was found between inferior and temporal GCC and myopic SE; but a significant moderate negative correlation was found between all sectors of GCC and hyperopic SE. For GCIPL, significant weak to moderate positive correlations were found with myopic SE for all sectors. The summary of the correlations between GCC and GCIPL with refraction groups is shown in Table 7.

Line 296: Table 7. Correlation between GCC and GCIPL with SE groups

Discussion: Line 466-470

Response to Riewer #2

While there have been similar studies conducted in different populations, like Chinese, Tibetan, German, Romanian etc, this aims to give a reference database in the Malaysian paediatric population. If possible, the authors can briefly summarise the average findings of the various studies in different populations in the form of a table.

Our response: The average findings of various studies have been added to the tables and discussed in the discussion part.

RNFL: Discussion at Line 341-349

Line 350-351: Table 8. Comparison of pediatric RNFL thickness reported in the literature with the current study.

Macular: Discussion at Line 398-405

Line 406-407: Table 9. Comparison of pediatric average macular thickness and macular volume reported in the literature with the current study.

GCC & GCIPL: Discussion at Line 454-459

Line 460-461: Table 10. Comparison of pediatric GCC and GCIPL thickness reported in the literature with the current study.
